# 

**DOI:** 10.1186/1617-9625-3-1-1

**Published:** 2005-12-15

**Authors:** J Elliott Scott

**Affiliations:** 1 Departments of Oral Biology & Anatomy, University of Manitoba, (Winnipeg, Manitoba, Canada)

## 

With the death of ABC journalist Peter Jennings during the summer from lung cancer, apparently from smoking over many years, the media particularly in the United States and to some extent in Canada as Jennings was a Canadian by birth, has seen fit to extend their coverage to the many anti-smoking initiatives in both countries. While the intent to highlight the harm done by smoking was obvious, the coverage did little to support the views of the medical community that long term tobacco smoking and for that matter, inhalation of second hand tobacco smoke, is an established risk factor for anything other than lung cancer.

Indeed suggestions that little or no progress has been made in diagnosis and treatment of this or other tobacco-smoke linked cancers seemed a bit disturbing. This coverage included physicians involved in clinical anti-smoking campaigns, the occasional basic medical scientist and epidemiologists studying trends in smoking habits, particularly in teenagers. (see article by von Ah in this issue). Yet among these professional anti-smoking groups, an interview with a Philip Morris executive stood out as particularly disturbing. This related to the continuing belief that tobacco companies are still targeting teenagers and younger children to replenish the progressively declining numbers of adult smokers. The specious arguments from these officials that the use of cartoon-like characters in advertisements does not directly target young potential smokers seems amazingly duplicitous. So it would seem that despite multimillion dollar settlement suits against the tobacco companies, little has changed in their perception of risk-harm assessments. This demonstration of the continuing belief of the tobacco companies that the public accepts their statements at face value, even after decades of misrepresentation, is amazing for those of us who work in the various aspect of research related to tobacco smoke and have some knowledge of the well documented harm caused by smoking. Sadly the tobacco companies' views probably represent the majority views of the public at large and in many respects is therefore very accurate. This raises the question as to why this should be so. Two rationalé come immediately to mind. Social acceptance of smoking was, at least some years ago, unquestioned. In many parts of the world this situation remains. In addition, the detrimental health effects of smoking are neither readily apparent nor immediate in their demonstrability. Indeed with the exception of lung cancer, a good percentage of the public would still seem to view the issue of the detrimental effects of tobacco smoking as unproven. Indeed the long latency of tobacco effects and in fact the resiliency of biological model systems has become quite clear in experiments ongoing in our and other laboratories. This should not be construed however to suggest that smoking or exposure to ETS is benign for it is very difficult in the laboratory to emulate the *in vivo *exposures of many years to the complex array of carcinogens in smoke. Indeed new molecular approaches to cellular changes induced by single or multiple components of smoke have only begun to characterize mechanisms whereby these agents may activate and alter cellular metabolic processes.

New research into the many risks associated with tobacco smoke exposure requires many forums for investigation, public exposure and review. Without a doubt, smoking induces changes at the cellular and molecular levels in many organs. From initial exposure via the huge surface area of the pulmonary tissues which is often unappreciated, circulatory distribution of dozens of toxins has effects, largely unknown, on many tissues, probably related to each individual tissue's spectrum of susceptibility and expression of receptors for the numerous toxins as well as toxin lipid solubility. The grounds for further research into these effects is a very fertile area and the complexity of these potential interactions will be without a doubt very difficult to interpret and controversial.

Taking on these many challenges, the journal has identified some five areas which will be pursued. Four of these are already well under way, largely due to the pioneering efforts of Dr. Longo, who has established the scientific integrity of this journal. This will soon be reflected in a move to BioMed Central. Without his efforts we could not hope to continue further publications and our efforts to establish a reputable scientific publication. The fifth and final area must be approached very cautiously due to the somewhat controversial and difficult nature of research in the area. This is of course research into cellular and molecular changes induced by smoke and smoke components in the many organ systems which are well established to be influenced via direct exposure such as the lung or through distribution by the circulatory system. We hope to establish this pillar of biomedical research as a significant component of our publication. Integrity in this and the other areas will of course come from quality publications and international recognition of the editorial board on whom we will all rely heavily. The support of the members of the editorial board and executive of the ISPTID is hereby gratefully acknowledged.

Finally we must again acknowledge the efforts of Dr. Daniel Longo in providing direction and insight in the initial phases of development of the journal. We would encourage all researchers in areas related to smoking epidemiology or biomedical application to consider the journal for publication of their results.

## Competing interests

The authors declare that they have no competing interests.

